# Robotic versus laparoscopic inguinal hernia repair: an updated systematic review and meta-analysis

**DOI:** 10.1007/s11701-021-01312-6

**Published:** 2021-10-05

**Authors:** Leonardo Solaini, Davide Cavaliere, Andrea Avanzolini, Giuseppe Rocco, Giorgio Ercolani

**Affiliations:** 1 Department of Medical and Surgical Sciences (DIMEC), University of Bologna, Forlì, Italy; 2 General and Oncologic Surgery, Morgagni-Pierantoni Hospital, Via Forlanini 34, Forlì, FC Italy

**Keywords:** Groin hernia repair, Minimally invasive repair, Robotic surgery, Robotic hernia surgery

## Abstract

The aim of this study was to review the latest evidence on the robotic approach (RHR) for inguinal hernia repair comparing the pooled outcome of this technique with those of the standard laparoscopic procedure (LHR). A systematic literature search was performed in PubMed, Web of Science and Scopus for studies published between 2010 and 2021 concerning the comparison between RHR versus LHR. After screening 582 articles, 9 articles with a total of 64,426 patients (7589 RHRs) were eligible for inclusion. Among preoperative variables, a pooled higher ratio of ASA > 2 patients was found in the robotic group (12.4 vs 8.6%, *p* < 0.001). Unilateral hernia repair was more common in the laparoscopic group (79.9 vs 68.1, *p* < 0.001). Overall, operative time was longer in the robotic group (160 vs 90 min, *p* < 0.001); this was confirmed also in the sub-analysis on unilateral procedures (88 vs 68 min, *p* = 0.040). The operative time for robotic bilateral repair was similar to the laparoscopic one (111 vs 100, *p* = 0.797). Conversion to open surgery was 0% in the robotic group. The pooled rate of chronic pain and postoperative complications was similar between the groups. The standardized mean difference MD of the costs between LHR versus RHR was − 3270$ (95% CI – 4757 to − 1782, *p* < 0.001). In conclusion, laparoscopic and robotic inguinal hernia repair have similar safety parameters and postoperative outcomes. Robotic approach may require longer operative time if the unilateral repair is performed. Costs are higher in the robotic group.

## Introduction

The first inguinal hernia repair with the robotic approach dates back to 2007 [1]. Since then, there have been a growing interest on exploiting the robotic platform for repairing abdominal wall defects. As such, the search term “robotic inguinal hernia repair” on Pubmed have been yielding a constant increase in the results since its first appearance in the MEDLINE database.

Few comparative studies have been published, highlighting the differences between the minimally invasive and the traditional open approach [2]. Based on these results the European Hernia Society’s international guidelines recommended laparoscopic inguinal hernia repair for improved postoperative pain outcomes compared with open surgery [3].

The technology offered by the robotic platform, such as magnified 3-dimensional visualization, stable platform and seven degrees of freedom wrists, have been reported to improve clinical outcomes and to ease the procedure for the surgeon. As per inguinal hernia repair, these advantages may reflect an improved visualization of the inguinal anatomy and easier and more accurate dissections.

However, only a few studies comparing the laparoscopic and the robotic approach have been published [4–16]. The majority of them were characterized by small sample size, especially in the robotic arm, [6, 7, 9, 16, 17], limiting the power and, thus, the generalizability, of the studies.

Recently three meta-analyses have been published on the topic [18–20] and the most recent is updated until May 2020. Since then, several studies on the comparison between the robotic and the laparoscopic approach to repair inguinal hernia have been published adding further evidence on the topic. They included the first randomized controlled trial [14] and the two largest comparative studies [8, 15] for a total of 6670 additional robotic inguinal hernia repairs which could be analyzed for more representative pooled outcomes.

The aim of this study was to review the latest evidence on the robotic approach for inguinal hernia repair comparing the pooled outcome of this technique with those of the standard laparoscopic procedure.

## Methods

### Study selection

This study was performed adhering to the 2010 Preferred Reporting Items for Systematic Reviews and Meta-Analyses (PRISMA) guidelines [21] and to AMSTAR (Assessing the methodological quality of systematic reviews) Guidelines [22]. A systematic literature search was performed in PubMed, Embase, and Scopus for studies published between January 1st, 2010, and July 29th, 2021. Search terms used were *robotic inguinal hernia repair*. Titles, abstracts and full-text articles were screened and selected by two authors (LS and GR) independently based on eligibility criteria. Disagreement on eligibility was addressed by discussion until consensus was obtained.

### Eligibility criteria and assessment of methodological quality

Comparative studies in English concerning robotic versus laparoscopic inguinal hernia repair that were available in full-text were included. For institutions reporting overlapping data, only the study with the largest number of robotic procedure was included. Excluded were abstracts, case reports, editorials, reviews and studies with no data on the outcomes of interest or with less than 40 robotic procedures.

Methodological quality was assessed by two authors (LS and GR) using the Newcastle–Ottawa Scale [23] for cohort studies and the Jadad scoring for randomized controlled trials [24].

### Data extraction

Data extracted included study characteristics (country of origin, study period, study design, sample size), patients’ characteristics (age, sex, BMI) and operative (operative time, conversion to open surgery) and postoperative outcomes (overall and detailed complications, 90-day re-admission). The postoperative complication rate was the primary outcomes.

### Statistical analysis

For categorical variables, the weighted pooled rates with 95% confidence intervals (95% CI) were calculated exploiting the Freeman–Tukey transformation [25]. Continuous variables were pooled in weighted means and 95% CI using the inverse variance method. Continuous variables as median and interquartile range (or median and range) were transformed in mean and standard deviation (SD) as suggested by Hozo et al. [26]. The relative risk (RR) or the standardized mean difference (SMD) was calculated when required according to the type of variable (continuous/dichotomous) analyzed. Heterogeneity among the included studies was verified by inconsistency (*I*^2^) statistics [27] and, if present, the random effects model was used. Statistical analyses were performed with MedCalc Statistical Software version 15.8 (MedCalc Software bvba, Ostend, Belgium; https://www.medcalc.org; 2015).

## Results

### Literature search results

**Fig. 1:**
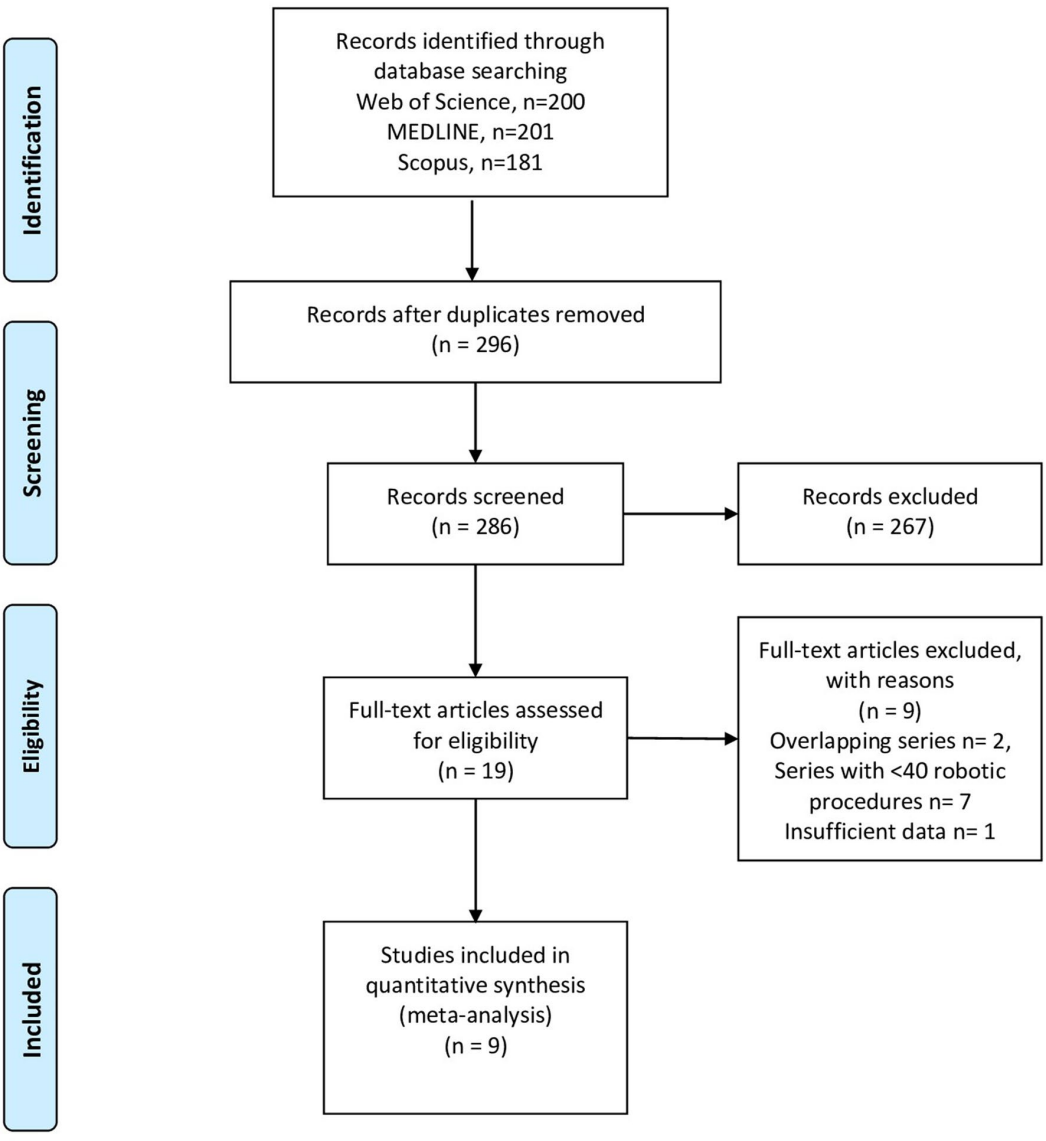
PRISMA flowchart

Literature search yielded a total of 482 potentially relevant articles (Fig. 1). Of these, nine studies published between 2010 and 2021 were found to be eligible for data extraction and were therefore included in the meta-analyses [4, 5, 8, 10–15]. A total of 62,426 patients who underwent RHR (*n* = 7589) or LHR (*n* = 54,837) from 2010 to 2021 were identified. The quality assessments for each study are summarized in Table 1.

**Table 1:** Study characteristics and quality assessment

First author	Country	Study period	Study design	Technique used	Robot version	Robotic (*n*)	Laparoscopic	Bilateral (*n*)	Quality assessment
Kudsi [11]	USA	2012–2015	Retrospective, single center	L-TEP/R-TAPP	Da Vinci Si	118	157	37L/35R	8/9
Charles [5]	USA	2012–2016	Retrospective, single center	L-TAPP/R-TAPP	n.a	69	241	0/0	5/9
Muysoms [12]	Belgium	2013–2017	Prospective, single center	L-TAPP/R-TAPP	Da Vinci Xi	49	64	42L/15R	7/9
Pokala [13]	USA	2013–2017	Retrospective, multicenter	n.a	n.a	594	540	n.a	5/9
Prabhu [14]	USA	2016–2019	Prospective randomized trial, multicenter	L-TAPP/R-TAPP	n.a	48	54	0/0	3/5
Khoraki [10]	USA	2015–2017	Retrospective, single center	L-TEP/R-TAPP	Da Vinci	45	138	41L/8R	5/9
Aghayeva [4]	Turkey	2016–2018	Retrospective, single center	L-TEP/R-TAPP	Da Vinci Xi	43	43	22L/22R	8/9
Tatarian [15]	USA	2010–2016	Retrospective, multicenter	n.a	n.a	559	35,565	n.a	6/9
Holleran [8]	USA	2008–2019	Retrospective multicenter	n.a	n.a	6063	18,035	n.a	5/9

*L-* laparoscopic, *R-* robotic, *TEP* totally extraperitoneal approach, *TAPP* transabdominal preperitoneal approach, *n.a.* detail not available

### Characteristics of the studies

Details of included study are reported in Table 1. Only one multicenter randomized controlled trial was included [14]. One study analyzed the data which were extracted from an administrative database of the state of New York [15]; two studies exploited quality improvement databases [5, 8] while Pokala et al. queried a multi-institutional clinical database [13]. The remaining four studies were single-center comparative analyses. Operative time and any postoperative occurrence or recurrence/groin pain were the primary outcomes of three studies [4, 5, 12]; this was not selected in the study by Prabhu et al. as it was designed as a pilot study [14]. In six studies, the surgical technique to repair inguinal hernia was specified [4, 5, 10–12, 14]: a totally extraperitoneal approach (TEP) was used only in the laparoscopic arms in three studies [4, 10, 11] while the robotic procedures were performed via a transabdominal preperitoneal approach (TAPP) in all cases.

In five studies, details regarding the mesh used were reported [4, 10–12, 14]. Two authors [11, 12] used a laparoscopic self-fixating mesh. A flat polypropylene mesh was used for all repairs in the study by Prabhu et al. [14]. A prosthetic 3DMax™ mesh was adopted by Aghayeva et al. [4] for both approaches. Khoraki et al. used Parietex™ and 3DMax™ meshes in both laparoscopic and robotic repairs [10].

Only three studies reported a follow-up of at least 1 year to evaluate late complications or recurrence [4, 11, 15]. The remaining six studies [5, 8, 10, 12–14] reported short-term outcomes within the 30th postoperative day.

### Patients’ characteristics and perioperative outcomes

Patients’ characteristics are reported in Table 1. Among preoperative variables, a pooled higher ratio of ASA > 2 patients was found in the robotic group (12.4 vs 8.6%, *p* < 0.001). Unilateral hernia repair was more common in the laparoscopic group (79.9 vs 68.1, *p* < 0.001).

Overall, operative time was longer in the robotic group (160 vs 90 min; *p* < 0.001); this was confirmed also in the sub-analysis on unilateral procedures (88 vs 68 min; *p* = 0.040). The operative time for robotic bilateral repair were similar to the laparoscopic one (111 vs 100 min, SMD 0.1, − 0.3 to 0.4; *p* = 0.797).

Conversion to open surgery was absent in the robotic group; only Prabhu et al. reported a conversion to the laparoscopic approach due to bleeding [14]. Two studies reported one case of conversion to open procedure in the laparoscopic group [10, 11].

Five articles reported data on postoperative pain [4, 11–14] which was similar between the groups. Prabhu et al. used the Visual Analog Scale to quantify the postoperative pain while Muysoms et al. used the EuraHS Quality of Life Questionnaire [12, 28]. Pokala et al. used the need of opioids in the postoperative period as surrogate marker. The pooled rate of chronic pain, which was calculated only from two studies [4, 11] was similar between the groups (Table 2).

**Table 2:** Pooled analyses of robotic versus laparoscopic inguinal hernia repair

Variable	No. of patients	Robotic	Laparoscopic	*P*	*I*^2^ (95% CI)	References
Age (years)	61,292	57.4 (53.9–60.7)	55.5 (55.2–58.8)	0.669	96.0 (94.0–97.4)	[4, 5, 8, 10–12, 14, 15]
ASA > 2 (%)	24,952	12.4 (3.2–26.4)	8.6 (1.8–19.9)	< 0.001	53.5 (0.0–82.9)	[4, 5, 8, 10, 11]
Sex—f—(%)	62,426	8.2 (6.4–10.4)	7.0 (2.9–12.6)	0.580	98.0 (97.2–98.5)	[4, 5, 8, 10–15]
BMI	24,168	26.5 (24.3–28.8)	25.88 (25.5–26.3)	0.743	96.3 (94.3–97.6)	[4, 8, 10–12, 14]
Bilateral (%)	1070	16.8 (3.5–37.0)	21.8 (3.8–49.0)	0.324	75.9 (33.6–91.2)	[4, 5, 11, 12, 14]
Unilateral (%)	463	68.1 (30.1–95.6)	79.9 (46.5–98.7)	< 0.001	10.1 (0.0–97.0)	[4, 11, 14]
Operative time (min)	24,184	160 (99–222)	90 (89–90)	< 0.001	87.2 (50.0–96.7)	[4, 8]
Bilateral	458	111 (49–173)	100 (75–125)	0.797	25.8 (0.0–0.0)	[10, 11]
Unilateral	687	88 (69–108)	68 (44–92)	0.047	98.9 (98.1–99.3)	[5, 11, 14]
Conversion to open surgery (%)	760	0.4 (0.0–1.4)	4.1 (3.0–5.6)	0.702	0.0 (0.0–0.0)	[4, 10–12, 14]
Complications (%)	62,426	8.9 (5.4–13.5)	4.2 (3.0–5.6)	0.097	92.7 (88.3–95.4)	[4, 5, 8, 10–15]
Urinary retention (%)	1070	2.7 (1.3–4.8)	2.5 (0.8–5.1)	0.943	0.0 (0.0–0.0)	[4, 5, 10–12, 14]
Seroma/hematoma (%)	1070	6.1 (3.9–9.0)	5.1 (1.1–11.8)	0.154	0.0 (0.0–46.1)	[4, 5, 10–12, 14]
Chronic pain (%)	361	2.1 (0.5–5.7)	1.3 (0.2–4.0)	0.565	0.0 (0.0–0.0)	[4, 11]
30-Day re-admission (%)	38,214	3.2 (1.1–6.4)	1.6 (1.0–2.4)	0.339	83.3 (65.0–92.0)	[4, 5, 10, 11, 13–15]
30-Day mortality (%)	26,086	0.1 (0.1–0.2)	0.1 (0.0–0.1)	0.337	0.0 (0.0–91.3)	[4, 5, 8, 10, 11, 13]
1-Year recurrence (%)	36,485	1.6 (0.8–2.7)	0.9 (0.8–1.0)	0.067	0.0 (0.0–0.0)	[4, 11, 15]
Discharged on the same day (%)	699	80.6 (27.6–98.9)	89.4 (57.3–99.8)	0.421	96.1 (91.6–98.2)	[4, 5, 12]
Hospital stay (days)	61,625	1.8 (0.4–3.3)	1.2 (0.8–1.7)	0.195	99.3 (98.9–99.5)	[4, 8, 10, 13, 15]

Data about total hospital costs could be extracted from four studies [4, 10, 13, 14]. The SMD of the costs between LHR versus RHR was − 3270$ (95% CI – 4757 to − 1782, *p* < 0.001).

## Discussion

Robotic inguinal hernia repair has outcomes similar to the laparoscopic approach. Overall complications, chronic postoperative pain, urinary retention and 30-day re-admission were superimposable between the groups.

As it has been reported for other fields of general surgery [29–32], the robotic approach required longer operative times also for inguinal hernia repair. This has been also confirmed in two recent meta-analyses [19, 20], which reported only the overall operative duration including both bilateral and unilateral repairs. However, especially in the present study, overall pooled mean operative time may be influenced by the different rate of unilateral hernia repair performed with the two approaches. Still, the pooled mean operative time of the unilateral repair showed a mean difference of + 20 min between the robotic and the laparoscopic approach. This additional time may represent the docking/de-docking time required in the robotic procedures. Interestingly, this difference seemed to be leveled in the bilateral repair and this might suggest a shorter surgical time with the robotic approach in this setting.

The type of prosthesis used to repair the defect was different among the studies reporting this detail [4, 10–12, 14]. This may strongly influence early outcomes of inguinal hernia repair [33] beyond the type of approach used and should be taken into account when interpreting the results of these studies. Recently, Muysoms et al. [34] reported promising outcomes on the use of one large self-fixating mesh to be used during laparoscopic bilateral groin hernia repair. In our opinion, this type of mesh may be particularly of use with the robotic approach which could take advantage of the additional degrees of freedom of the robotic wrist for an easier fixation and, thus, a potentially reduced operative time.

Postoperative pain, chronic pain, or inguinodynia were heterogeneously reported by the included studies, but no differences were found between the two approaches. Again, no distinctions were made to identify those cases of pain according to the type of procedure (bilateral/unilateral) and this might have had an impact on the results. In addition, most of the included studies were characterized by a 30-day follow-up which may be too short to define chronic pain.

To overcome these differences in reporting outcome a standard definition should be identified in order to perform more accurate and comparable analyses. As such, the use of a national/international registry may be advocated and it may help in limiting the entry of heterogeneous data by using standardized definitions [35].

Our analyses suggested no differences on recurrence according to the approach chosen for inguinal hernia repair. However, only three of the included studies reported a follow up longer than 30 days [4, 11, 15] with the most recent guidelines which recommended study follow-up of 3–5 years [3]. It must be also noted that, as it occurred for other variables, it could not be possible to extract the recurrence rate according to the type of procedure (bilateral/unilateral). In our opinion, this is a factor which should always be specified, especially in light of the higher rate of bilateral repair with the robotic procedures.

No data with regard to patients who had previous prostatectomies were reported in the included studies. Recent papers showed that the robotic platform with its improved dexterity and enhanced high-definition three-dimensional image may be particularly helpful in the challenging dissection of the retro-pubic space in patients who had prior prostatectomies [36, 37]. Those advantages offered by the robotic platform may expand the indications for minimally invasive inguinal hernia repair also for those cases which, nowadays, are performed only by expert laparoscopic surgeons. Still, large studies reporting on these patients are needed to confirm the benefit of the robotic approach in this particular setting.

This study has few limitations. First, the pooled outcomes may reflect the risk of selection bias of the majority of the included studies, as only one randomized clinical trial was included. Second, the results do not consider the learning curve in both groups, which may have affected the outcomes of the robotic approach in at least one study [12]. Third, details on the technique used to repair the inguinal hernia were reported only in six out of nine studies and three of them compared the laparoscopic totally extraperitoneal (TEPP) approach versus the robotic transabdominal preperitoneal (TAPP). This intra-study heterogeneity was a major limitation which may have contributed in not highlighting one approach rather than another. Fourth, an appropriate cost–benefit analysis was performed in no studies and these aspects should never be underestimated when dealing with highly expensive technology such as the robotic platform. Finally, it must be considered that five out of nine studies [5, 11–14] including the randomized controlled trial were conducted by authors who stated financial ties with companies who are in the surgical robot market.

In conclusions, laparoscopic and robotic inguinal hernia repair have similar safety parameters and postoperative outcomes. Robotic approach may require longer operative time if the unilateral repair is performed. Further studies focusing only on bilateral repair may help highlighting the advantages on using the robotic platform.

## Data Availability

Data available upon reasonable request.
